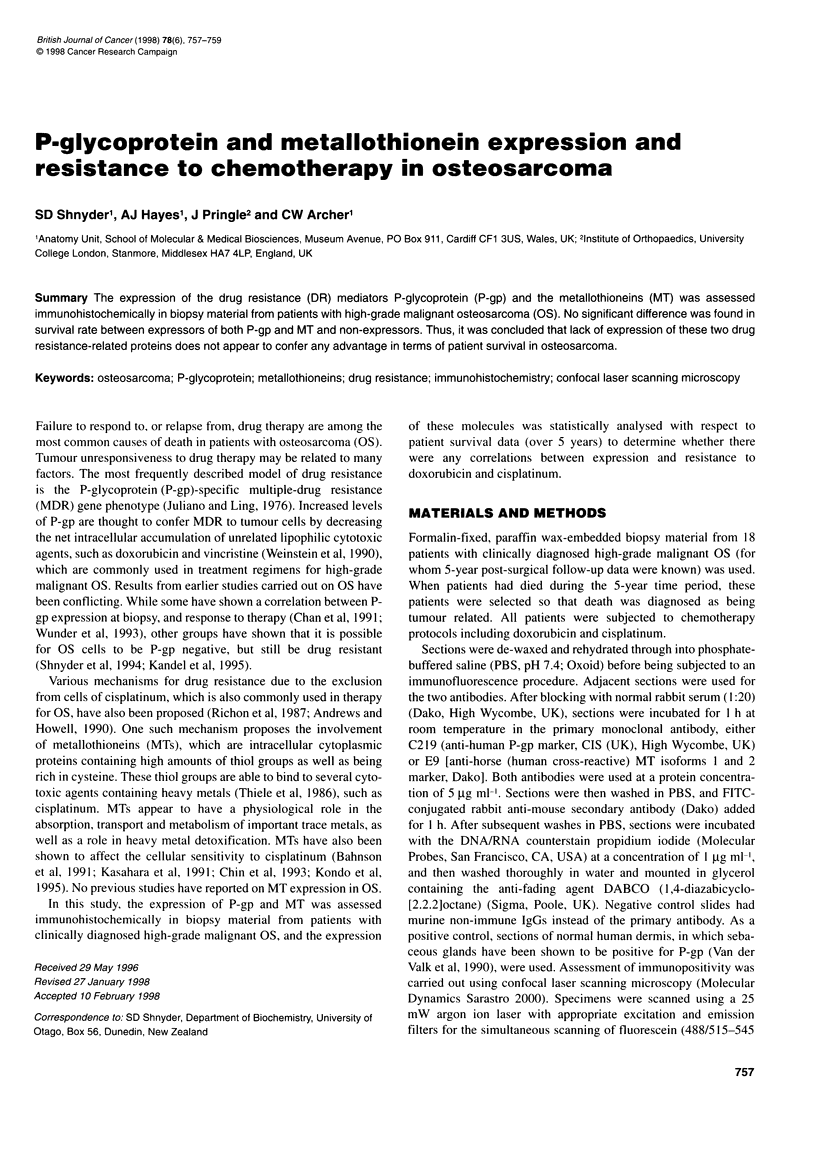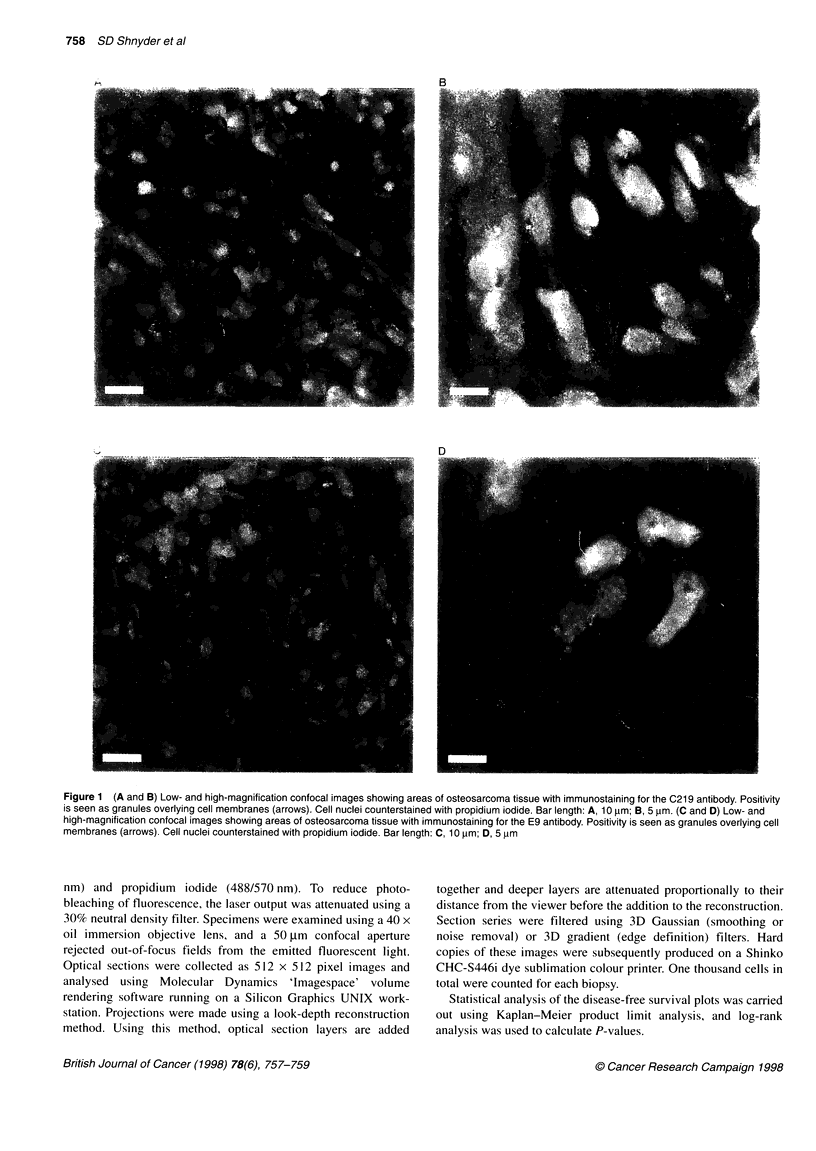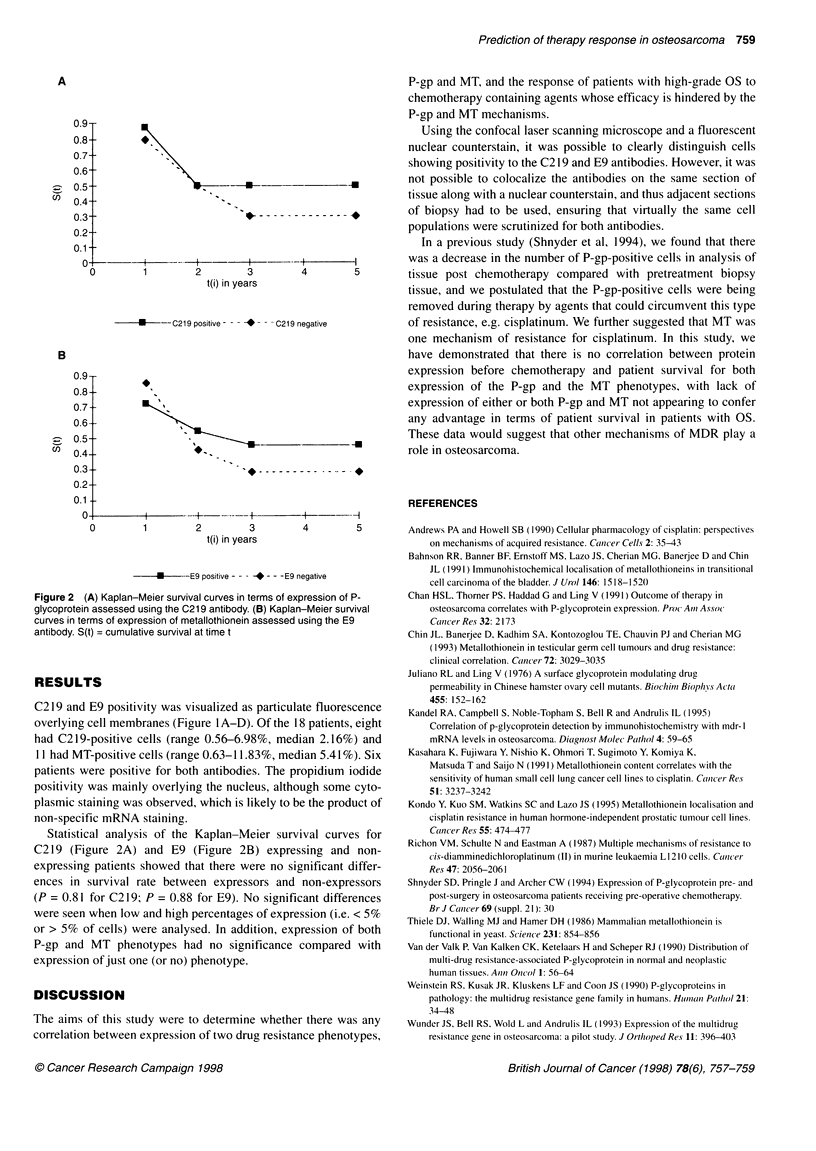# P-glycoprotein and metallothionein expression and resistance to chemotherapy in osteosarcoma.

**DOI:** 10.1038/bjc.1998.573

**Published:** 1998-09

**Authors:** S. D. Shnyder, A. J. Hayes, J. Pringle, C. W. Archer

**Affiliations:** Anatomy Unit, School of Molecular & Medical Biosciences, Cardiff, Wales, UK.

## Abstract

**Images:**


British Journal of Cancer (1998) 78(6), 757–759
© 1998 Cancer Research Campaign

### P-glycoprotein and metallothionein expression and resistance to chemotherapy in osteosarcoma

**SD Shnyder[1], AJ Hayes[1], J Pringle[2] and CW Archer[1]**

[1]Anatomy Unit, School of Molecular & Medical Biosciences, Museum Avenue, PO Box 911, Cardiff CF1 3US, Wales, UK; [2]Institute of Orthopaedics, University College London, Stanmore, Middlesex HA7 4LP, England, UK

**Summary** The expression of the drug resistance (DR) mediators P-glycoprotein (P-gp) and the metallothioneins (MT) was assessed immunohistochemically in biopsy material from patients with high-grade malignant osteosarcoma (OS). No significant difference was found in survival rate between expressors of both P-gp and MT and non-expressors. Thus, it was concluded that lack of expression of these two drug resistance-related proteins does not appear to confer any advantage in terms of patient survival in osteosarcoma.

**Keywords:** osteosarcoma; P-glycoprotein; metallothioneins; drug resistance; immunohistochemistry; confocal laser scanning microscopy

Failure to respond to, or relapse from, drug therapy are among the most common causes of death in patients with osteosarcoma (OS). Tumour unresponsiveness to drug therapy may be related to many factors. The most frequently described model of drug resistance is the P-glycoprotein (P-gp)-specific multiple-drug resistance (MDR) gene phenotype (Juliano and Ling, 1976). Increased levels of P-gp are thought to confer MDR to tumour cells by decreasing the net intracellular accumulation of unrelated lipophilic cytotoxic agents, such as doxorubicin and vincristine (Weinstein et al, 1990), which are commonly used in treatment regimens for high-grade malignant OS. Results from earlier studies carried out on OS have been conflicting. While some have shown a correlation between P-gp expression at biopsy, and response to therapy (Chan et al, 1991; Wunder et al, 1993), other groups have shown that it is possible for OS cells to be P-gp negative, but still be drug resistant (Shnyder et al, 1994; Kandel et al, 1995).

Various mechanisms for drug resistance due to the exclusion from cells of cisplatinum, which is also commonly used in therapy for OS, have also been proposed (Richon et al, 1987; Andrews and Howell, 1990). One such mechanism proposes the involvement of metallothioneins (MTs), which are intracellular cytoplasmic proteins containing high amounts of thiol groups as well as being rich in cysteine. These thiol groups are able to bind to several cytotoxic agents containing heavy metals (Thiele et al, 1986), such as cisplatinum. MTs appear to have a physiological role in the absorption, transport and metabolism of important trace metals, as well as a role in heavy metal detoxification. MTs have also been shown to affect the cellular sensitivity to cisplatinum (Bahnson et al, 1991; Kasahara et al, 1991; Chin et al, 1993; Kondo et al, 1995). No previous studies have reported on MT expression in OS.

In this study, the expression of P-gp and MT was assessed immunohistochemically in biopsy material from patients with clinically diagnosed high-grade malignant OS, and the expression of these molecules was statistically analysed with respect to patient survival data (over 5 years) to determine whether there were any correlations between expression and resistance to doxorubicin and cisplatinum.

*Received 29 May 1996*
*Revised 27 January 1998*
*Accepted 10 February 1998*

*Correspondence to:* SD Shnyder, Department of Biochemistry, University of Otago, Box 56, Dunedin, New Zealand

### MATERIALS AND METHODS

Formalin-fixed, paraffin wax-embedded biopsy material from 18 patients with clinically diagnosed high-grade malignant OS (for whom 5-year post-surgical follow-up data were known) was used. When patients had died during the 5-year time period, these patients were selected so that death was diagnosed as being tumour related. All patients were subjected to chemotherapy protocols including doxorubicin and cisplatinum.

Sections were de-waxed and rehydrated through into phosphate-buffered saline (PBS, pH 7.4; Oxoid) before being subjected to an immunofluorescence procedure. Adjacent sections were used for the two antibodies. After blocking with normal rabbit serum (1:20) (Dako, High Wycombe, UK), sections were incubated for 1 h at room temperature in the primary monoclonal antibody, either C219 (anti-human P-gp marker, CIS (UK), High Wycombe, UK) or E9 [anti-horse (human cross-reactive) MT isoforms 1 and 2 marker, Dako]. Both antibodies were used at a protein concentration of 5 $\mu g\ ml^{-1}$. Sections were then washed in PBS, and FITC-conjugated rabbit anti-mouse secondary antibody (Dako) added for 1 h. After subsequent washes in PBS, sections were incubated with the DNA/RNA counterstain propidium iodide (Molecular Probes, San Francisco, CA, USA) at a concentration of 1 $\mu g\ ml^{-1}$, and then washed thoroughly in water and mounted in glycerol containing the anti-fading agent DABCO (1,4-diazabicyclo-[2.2.2]octane) (Sigma, Poole, UK). Negative control slides had murine non-immune IgGs instead of the primary antibody. As a positive control, sections of normal human dermis, in which sebaceous glands have been shown to be positive for P-gp (Van der Valk et al, 1990), were used. Assessment of immunopositivity was carried out using confocal laser scanning microscopy (Molecular Dynamics Sarastro 2000). Specimens were scanned using a 25 mW argon ion laser with appropriate excitation and emission filters for the simultaneous scanning of fluorescein (488/515–545

A B C D

**Figure 1** (**A** and **B**) Low- and high-magnification confocal images showing areas of osteosarcoma tissue with immunostaining for the C219 antibody. Positivity is seen as granules overlying cell membranes (arrows). Cell nuclei counterstained with propidium iodide. Bar length: **A**, 10 μm; **B**, 5 μm. (**C** and **D**) Low- and high-magnification confocal images showing areas of osteosarcoma tissue with immunostaining for the E9 antibody. Positivity is seen as granules overlying cell membranes (arrows). Cell nuclei counterstained with propidium iodide. Bar length: **C**, 10 μm; **D**, 5 μm nm) and propidium iodide (488/570 nm). To reduce photobleaching of fluorescence, the laser output was attenuated using a 30% neutral density filter. Specimens were examined using a 40 × oil immersion objective lens, and a 50 μm confocal aperture rejected out-of-focus fields from the emitted fluorescent light. Optical sections were collected as 512 × 512 pixel images and analysed using Molecular Dynamics 'Imagespace' volume rendering software running on a Silicon Graphics UNIX workstation. Projections were made using a look-depth reconstruction method. Using this method, optical section layers are added together and deeper layers are attenuated proportionally to their distance from the viewer before the addition to the reconstruction. Section series were filtered using 3D Gaussian (smoothing or noise removal) or 3D gradient (edge definition) filters. Hard copies of these images were subsequently produced on a Shinko CHC-S446i dye sublimation colour printer. One thousand cells in total were counted for each biopsy.

Statistical analysis of the disease-free survival plots was carried out using Kaplan–Meier product limit analysis, and log-rank analysis was used to calculate *P*-values.

© Cancer Research Campaign 1998

**Figure 2** (**A**) Kaplan–Meier survival curves in terms of expression of P-glycoprotein assessed using the C219 antibody. (**B**) Kaplan–Meier survival curves in terms of expression of metallothionein assessed using the E9 antibody. S(t) = cumulative survival at time t

### RESULTS

C219 and E9 positivity was visualized as particulate fluorescence overlying cell membranes (Figure 1A–D). Of the 18 patients, eight had C219-positive cells (range 0.56–6.98%, median 2.16%) and 11 had MT-positive cells (range 0.63–11.83%, median 5.41%). Six patients were positive for both antibodies. The propidium iodide positivity was mainly overlying the nucleus, although some cytoplasmic staining was observed, which is likely to be the product of non-specific mRNA staining.

Statistical analysis of the Kaplan–Meier survival curves for C219 (Figure 2A) and E9 (Figure 2B) expressing and non-expressing patients showed that there were no significant differences in survival rate between expressors and non-expressors ($P = 0.81$ for C219; $P = 0.88$ for E9). No significant differences were seen when low and high percentages of expression (i.e. < 5% or > 5% of cells) were analysed. In addition, expression of both P-gp and MT phenotypes had no significance compared with expression of just one (or no) phenotype.

### DISCUSSION

The aims of this study were to determine whether there was any correlation between expression of two drug resistance phenotypes, P-gp and MT, and the response of patients with high-grade OS to chemotherapy containing agents whose efficacy is hindered by the P-gp and MT mechanisms.

Using the confocal laser scanning microscope and a fluorescent nuclear counterstain, it was possible to clearly distinguish cells showing positivity to the C219 and E9 antibodies. However, it was not possible to colocalize the antibodies on the same section of tissue along with a nuclear counterstain, and thus adjacent sections of biopsy had to be used, ensuring that virtually the same cell populations were scrutinized for both antibodies.

In a previous study (Shnyder et al, 1994), we found that there was a decrease in the number of P-gp-positive cells in analysis of tissue post chemotherapy compared with pretreatment biopsy tissue, and we postulated that the P-gp-positive cells were being removed during therapy by agents that could circumvent this type of resistance, e.g. cisplatinum. We further suggested that MT was one mechanism of resistance for cisplatinum. In this study, we have demonstrated that there is no correlation between protein expression before chemotherapy and patient survival for both expression of the P-gp and the MT phenotypes, with lack of expression of either or both P-gp and MT not appearing to confer any advantage in terms of patient survival in patients with OS. These data would suggest that other mechanisms of MDR play a role in osteosarcoma.

### REFERENCES

Andrews PA and Howell SB (1990) Cellular pharmacology of cisplatin: perspectives on mechanisms of acquired resistance. *Cancer Cells* **2**: 35–43

Bahnson RR, Banner BF, Ernstoff MS, Lazo JS, Cherian MG, Banerjee D and Chin JL (1991) Immunohistochemical localisation of metallothioneins in transitional cell carcinoma of the bladder. *J Urol* **146**: 1518–1520

Chan HSL, Thorner PS, Haddad G and Ling V (1991) Outcome of therapy in osteosarcoma correlates with P-glycoprotein expression. *Proc Am Assoc Cancer Res* **32**: 2173

Chin JL, Banerjee D, Kadhim SA, Kontozoglou TE, Chauvin PJ and Cherian MG (1993) Metallothionein in testicular germ cell tumours and drug resistance: clinical correlation. *Cancer* **72**: 3029–3035

Juliano RL and Ling V (1976) A surface glycoprotein modulating drug permeability in Chinese hamster ovary cell mutants. *Biochim Biophys Acta* **455**: 152–162

Kandel RA, Campbell S, Noble-Topham S, Bell R and Andrulis IL (1995) Correlation of p-glycoprotein detection by immunohistochemistry with mdr-1 mRNA levels in osteosarcoma. *Diagnost Molec Pathol* **4**: 59–65

Kasahara K, Fujiwara Y, Nishio K, Ohmori T, Sugimoto Y, Komiya K, Matsuda T and Saijo N (1991) Metallothionein content correlates with the sensitivity of human small cell lung cancer cell lines to cisplatin. *Cancer Res* **51**: 3237–3242

Kondo Y, Kuo SM, Watkins SC and Lazo JS (1995) Metallothionein localisation and cisplatin resistance in human hormone-independent prostatic tumour cell lines. *Cancer Res* **55**: 474–477

Richon VM, Schulte N and Eastman A (1987) Multiple mechanisms of resistance to *cis*-diamminedichloroplatinum (II) in murine leukaemia L1210 cells. *Cancer Res* **47**: 2056–2061

Shnyder SD, Pringle J and Archer CW (1994) Expression of P-glycoprotein pre- and post-surgery in osteosarcoma patients receiving pre-operative chemotherapy. *Br J Cancer* **69** (suppl. 21): 30

Thiele DJ, Walling MJ and Hamer DH (1986) Mammalian metallothionein is functional in yeast. *Science* **231**: 854–856

Van der Valk P, Van Kalken CK, Ketelaars H and Scheper RJ (1990) Distribution of multi-drug resistance-associated P-glycoprotein in normal and neoplastic human tissues. *Ann Oncol* **1**: 56–64

Weinstein RS, Kusak JR, Kluskens LF and Coon JS (1990) P-glycoproteins in pathology: the multidrug resistance gene family in humans. *Human Pathol* **21**: 34–48

Wunder JS, Bell RS, Wold L and Andrulis IL (1993) Expression of the multidrug resistance gene in osteosarcoma: a pilot study. *J Orthoped Res* **11**: 396–403

© Cancer Research Campaign 1998